# Multi-Omics Profiling Identifies Pathways Associated With CD8^+^ T-Cell Activation in Severe Aplastic Anemia

**DOI:** 10.3389/fgene.2021.790990

**Published:** 2022-01-04

**Authors:** Xing You, Qiong Yang, Kai Yan, Song-Rong Wang, Rong-Rong Huang, Shun-Qing Wang, Cai-Yue Gao, Liang Li, Zhe-Xiong Lian

**Affiliations:** ^1^ School of Biomedical Sciences and Engineering, South China University of Technology, Guangzhou International Campus, Guangzhou, China; ^2^ Chronic Disease Laboratory, School of Medicine, South China University of Technology, Guangzhou, China; ^3^ Guangdong Cardiovascular Institute, Guangdong Provincial People’s Hospital, Guangdong Academy of Medical Sciences, Guangzhou, China; ^4^ Department of Hematology, Guangzhou First People’s Hospital, The Second Affiliated Hospital of South China University of Technology, Guangzhou, China; ^5^ National Engineering Research Center for Tissue Restoration and Reconstruction, South China University of Technology, Guangzhou, China; ^6^ Key Laboratory of Biomedical Engineering of Guangdong Province, South China University of Technology, Guangzhou, China; ^7^ Key Laboratory of Biomedical Materials and Engineering of the Ministry of Education, South China University of Technology, Guangzhou, China; ^8^ Innovation Center for Tissue Restoration and Reconstruction, South China University of Technology, Guangzhou, China

**Keywords:** multi-omics, aplastic anemia, T cells, proteomics, metabolomics, single-cell RNA sequencing

## Abstract

Severe aplastic anemia (SAA) is an autoimmune disease characterized by immune-mediated destruction of hematopoietic stem and progenitor cells. Autoreactive CD8^+^ T cells have been reported as the effector cells; however, the mechanisms regulating their cell activation in SAA remain largely unknown. Here, we performed proteomics and metabolomics analyses of plasma and bone marrow supernatant, together with transcriptional analysis of CD8^+^ T cells from SAA patients and healthy donors, to find key pathways that are involved in pathogenic CD8^+^ T-cell activation. We identified 21 differential proteins and 50 differential metabolites in SAA patients that were mainly involved in energy metabolism, complement and coagulation cascades, and HIF-1α signaling pathways. Interestingly, we found that these pathways are also enriched in T cells from SAA patients by analyzing available single-cell RNA sequencing data. Moreover, CD8^+^ T cells from SAA patients contain a highly activated CD38^+^ subset, which was increased in the bone marrow of SAA patients and a murine model of SAA. This subset presented enriched genes associated with the glycolysis or gluconeogenesis pathway, HIF-1α signaling pathway, and complement associated pathways, all of which were of importance in T-cell activation. In conclusion, our study reveals new pathways that may regulate CD8^+^ T-cell activation in SAA patients and provides potential therapeutic targets for SAA treatment.

## Introduction

Severe aplastic anemia (SAA) is a bone marrow (BM) failure syndrome that is characterized by the destruction of hematopoietic stem cells (HSCs) and empty BM. Most patients respond to immunosuppressive therapies (ISTs), suggesting an immune mechanism of AA pathogenesis (Scheinberg and Young, 2012). However, about 30% of patients become tolerant to IST and need further therapies such as BM transplantation (Scheinberg et al., 2014, Rios et al., 2014). Increased BM accumulation of activated T cells that overexpress cytokines IFN-γ and TNF-α and decreased regulatory T cells are observed in AA patients, which are thought to mediate decreased self-renewal and even apoptosis of HSCs (Sloand et al., 2002, Kim et al., 2002; Young, 2018).

Among the T-cell subsets, autoreactive cytotoxic CD8^+^ T cells are key mediators of progressive BM failure, which inhibits hematopoiesis in AA (Hosokawa et al., 2016, Muranski et al., 2016; Giudice et al., 2018, Feng et al., 2018). Previous studies have demonstrated increased HLA-DR^+^ (Xing et al., 2014, Liu et al., 2014), CD57^+^ (Giudice et al., 2018, Feng et al., 2018), and CD27^+^ (Zhao et al., 2019, Zhang et al., 2019) subpopulations of CD8^+^ T cells in AA patients, indicating an activated phenotype of CD8^+^ T cells. However, the pathogenic mechanisms of CD8^+^ T-cell activation in SAA still need further elucidation.

AA is a systemic disease. Previous metabolomics and proteomics analyses revealed abnormal metabolite composition in AA patients (Zhong et al., 2015, Zhang et al., 2015; Hogan et al., 2019, Chini et al., 2019; Shao et al., 2021, Qi et al., 2021), which are involved in energy metabolism pathways. These studies partially showed the changed pathways in AA by single omics. While multi-omics analysis emerges as a new approach to understanding the pathogenesis of complex and heterogeneous diseases, particularly autoimmune diseases (Starchenko and Lauffenburger, 2018), it is promising to take this integrative approach to highlight interrelationships of molecules involved in this complex disease, AA. Previous studies have shown that systemic changes in metabolism and hormones can affect the function of immune cells, for example, T cells (Fan et al., 2019, Li et al., 2019). However, whether serum changes in metabolism and proteins are associated with CD8^+^ T-cell activation or the pathogenesis of AA development remains unknown.

In this study, we performed multi-omics analysis and established a possible link between plasma protein and metabolites changes and T-cell activation, which may contribute to SAA pathogenesis. We found that glycolysis or gluconeogenesis, cholesterol metabolism, and HIF-1α signaling pathways are closely related to T-cell activation, and we highlighted CD38^+^CD8^+^ T cells as a prognostic marker and a potential therapeutic target in AA.

## Materials and Methods

### Sample Collection

Anticoagulant peripheral blood and BM aspirate of SAA patients and healthy donors were obtained from Guangzhou First People’s Hospital. Patients were diagnosed with acquired AA according to the international AA Study Group Criteria (Marsh et al., 2009, Ball et al., 2009). Samples were centrifuged at 450 *g* for 5 min at 4°C; then plasma and BM supernatant were aliquoted and stored at −80°C until further processing.

### Sample Pre-processing

Proteins and metabolites were extracted as previously described (Dunn et al., 2011, Broadhurst et al., 2011; Yan et al., 2020, Da et al., 2020). Briefly, 450 μl of methanol (Optima™ liquid chromatography–mass spectrometry (LC-MS) grade) was added to 150 μl of plasma or BM supernatant. The mixtures were thawed on ice with shaking for 3 min. The mixed solutions were centrifuged at 14,000 *g* for 10 min at 4°C. A quarter of each supernatant was subjected to centrifugal freeze-drying (Beckman Allegra 64R, Beckman Coulter, Brea, CA, USA) and then dissolved in 100 μl of MeOH:H_2_O (v:v = 8:2; Pierce™ Water, LC-MS grade; Thermo Fisher Scientific, Waltham, MA, USA). After centrifugation at 14,000 *g* for 10 min at 4°C, the supernatant was collected for further detection.

### Liquid Chromatography/Mass Spectrometry Analysis

The metabolites in plasma and BM samples were separated on a high-performance liquid chromatography (HPLC) system (Thermo Fisher Scientific, Waltham, MA, United States) with a C18 column (100 mm × 2.1 mm, 1.7 μm, Thermo Fisher Scientific) at a flow rate of 0.25 ml min^−1^. Mobile phase A was 0.1% aqueous formic acid (v/v), and mobile phase B was methanol. Gradient elution was optimized as follows: 0–2 min, 2% B; 2–20 min, 2–100% B; 20–24 min, 100% B; 24–24.1 min, 100–2% B; and 24.1–30 min, 2% B. The column temperature was maintained at 35°C, and the injection volume was 5 µl.

The high-resolution MS for metabolite analysis was performed on a Thermo Scientific Q Exactive Plus mass spectrometry (Thermo Fisher Scientific, United States) with an electrospray ionization (ESI) source. Both negative and positive ion scan modes were carried out. The scan range was set at *m*/*z* 70–1,050 with an MS resolution of 70,000. The other MS parameters included the following: auxiliary gas flow, 10 arbitrary units; sheath gas flow, 35 arbitrary units; automatic gain control (AGC) target, 3e6 ions; capillary temperature, 320°C; S-lens radio frequency (RF), 50%; maximum injection time, 100 ms; and spray voltage, 3.5 kV.

Metabolites were identified by automatically comparing the precursor and product ions features (*m*/*z*) in the experimental samples with those of the chemical standards in the reference databases of Compound Discoverer 2.1.0.401 software (Thermo Fisher Scientific). The LC-MS/MS raw data were processed using Compound Discoverer 2.1 software to obtain the QC-normalized peak area. Then, the data were further processed in the MetaboAnalyst platform (https://www.metaboanalyst.ca/) including normalized by median and transformed by log(base 10) without scaling. Then, the normalized data were analyzed by partial least squares discriminant analysis (PLS-DA) and a volcano plot.

The samples for the proteomics study were processed and analyzed using the method and instrument parameters as described previously (Yan et al., 2020, Da et al., 2020), and proteins were identified using Proteomics Discovery Software (version 2.1, Thermo Fisher Scientific). Proteome data were analyzed in R (RStudio, Boston, MA, USA), and the pathway enrichment was analyzed using gseGO function in ClusterProfiler package (version 3.16.1) (Yu et al., 2012, Wang et al., 2012).

The original metabolome and proteome data can be found in the Supplementary Materials.

### Transcriptional Data Analysis

The publicly available single-cell RNA sequencing data (GSE145669) and microarray data (GSE3807) were obtained from the Gene Expression Omnibus (GEO). As for single-cell sequencing data quality control, cells that have more than 4,000 or less than 200 unique genes or have more than 25% mitochondrial genes were removed. Only genes expressed in 20 or more cells were used for further analyses. Then ribosome and mitochondria genes were filtered out. The pre-processed data matrix was transformed to a Seurat object using CreateSeuratObject function and normalized and log-transformed, and the log-transformed matrix was used for all downstream analyses in Seurat package (version 4.0.1). Principal component analysis (PCA) was used for dimensionality reduction using highly variable genes. With the use of the first fifteen principal components as input, the data in two dimensions were visualized with Uniform Manifold Approximation and Projection (UMAP). The gene set variation analysis (GSVA) and gene set enrichment analysis (GSEA) were performed using the GSVA package (version 1.36.3) and ClusterProfiler package (version 3.16.1), respectively. Gene sets were downloaded from the MSigDB database or collected from document literatures. The differences in pathway enrichment scores between different clusters were calculated using LIMMA package (version 3.44.3). As for microarray data analysis, the expression matrix was downloaded by GEOquery (version 2.10), and gene annotation was performed to transform the probe name to symbol; then expression matrix was normalized with log2. The normalized matrix was analyzed for differential expression, and then heatmap was obtained by pheatmap package (version 1.0.12).

### Aplastic Anemia Murine Model

C57BL/6 and BALB/c mice were purchased from Hunan SJA Laboratory Animal Co., Ltd (China). Male C57BL/6 mice were crossed with female BALB/c mice to generate CByB6F1 mice. To induce immune-mediated BM failure, CByB6F1 mice aged 8–12 weeks received 5 × 10^7^ mixed lymph node cells and splenocytes from age- and sex-matched C57BL/6 mice or phosphate-buffered saline (PBS) intravenously. Mice were sacrificed at 3–4 weeks after cell transfer. All mice were housed and bred in Laboratory Animal Research Center, South China University of Technology (SCUT), under a specific pathogen-free condition.

### Flow Cytometry

BM mononuclear cells were isolated, and red blood cells were depleted using red blood lysis buffer (Beyotime Biotechnology, Shanghai, China; C3702). For surface markers staining, cells were incubated with purified anti-CD16/CD32 antibody (BioLegend, San Diego, CA, USA) for 15 min at 4°C and then stained with fluorochrome-conjugated antibodies for 20 min at 4°C. The antibodies against Ter119 (TER-119), CD45 (104), CD11b (M1/70), CD19 (6D5), NK1.1 (PK136), CD3 (17A2), CD8α (53–6.7), FasL (MFL3), and CD44 (IM7) were purchased from BioLegend; the antibodies against CD4 (GK1.5) and CD62L (MEL-14) were purchased from BD Biosciences (BD Biosciences, San Jose, CA, USA). For intracellular cytokine staining, cells were resuspended in RPMI-1640 with 10% fetal bovine serum and stimulated with Cell Stimulation Cocktail (plus protein transport inhibitors) (eBioscience, San Diego, CA, United States) at 37°C for 3 h. After surface marker staining, cells were fixed and permeabilized with Cytofix/Cytoperm™ Fixation/Permeabilization Kit (BD Biosciences) and then stained for anti-IFN-γ and anti-TNF-α (BioLegend). Flow cytometric analysis was performed on LSR Fortessa (BD Biosciences), and data were analyzed with the Flowjo software (BD Biosciences).

### Statistics

Data were analyzed using R (RStudio, Boston, MA, United States) and GraphPad Prism 8 (GraphPad Software, San Diego, CA, USA). Results in all figures were expressed as mean ± standard error of the mean. For the comparison of two groups, if they pass homogeneity of variance test (*p* > 0.05), statistics were analyzed using a two-tailed unpaired *t*-test with Welch’s correction; otherwise, a nonparametric test was used. *p*-Values less than 0.05 were considered significantly different: ∗*p* < 0.05, ∗∗*p* < 0.01, and ∗∗∗*p* < 0.001.

## Results

### Proteomics Analysis of Plasma From Severe Aplastic Anemia Patients and Healthy Donors

**FIGURE 1:**
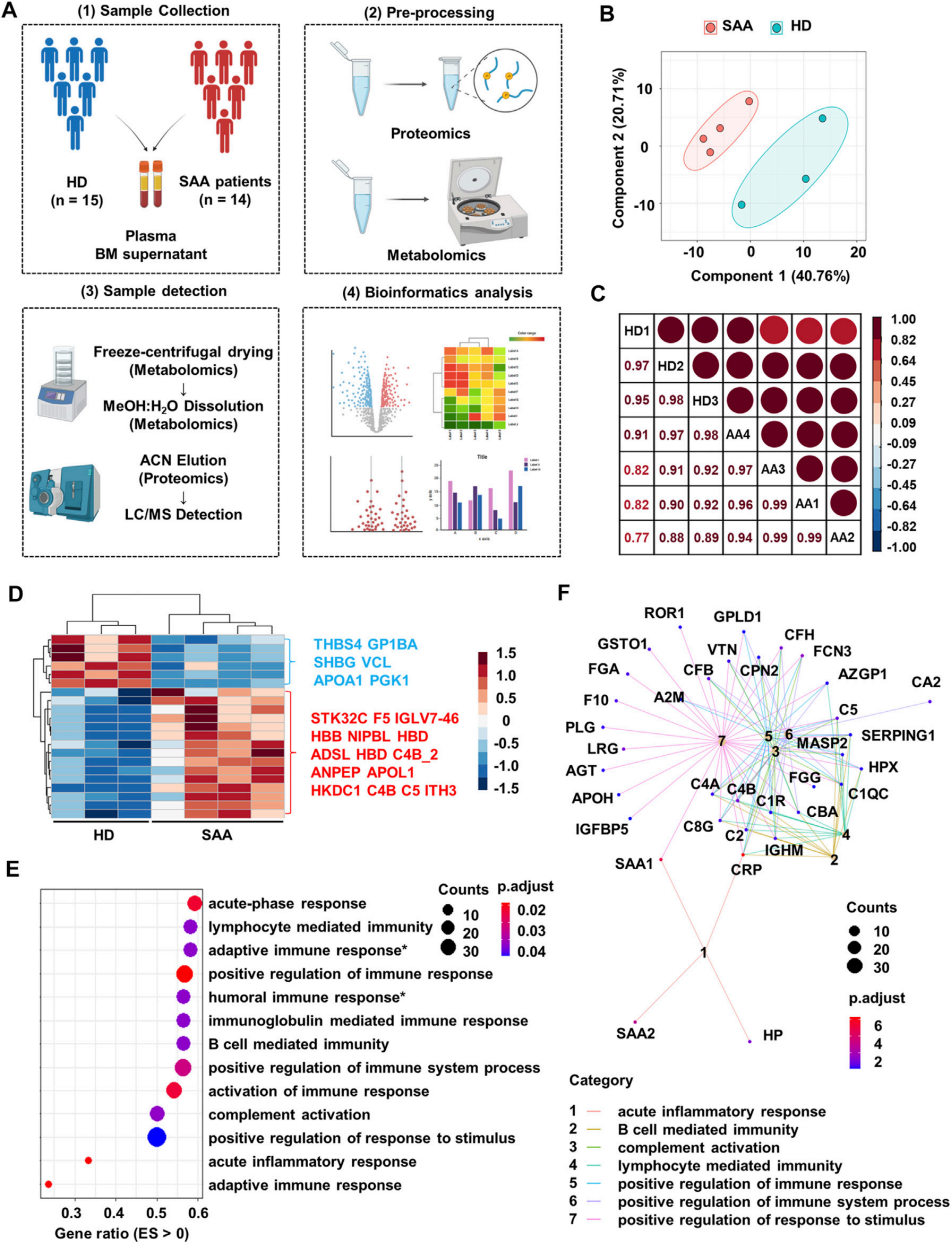
Proteomics profiling of plasma from SAA patients and healthy donors. **(A)** Schematic diagram of processes to obtain proteomics and metabolomics LC-MS of plasma and bone marrow supernatant from healthy donors (HD; *n* = 15) and SAA patients (SAA; *n* = 14). **(B)** PCA plot of the plasma protein samples from SAA (pink dots) patients and HD (green dots). **(C)** Pearson’s correlation analysis showing the coefficient of association of protein composition between each mixed sample from HD (*n* = 3) and SAA (*n* = 4). **(D)** Heatmap showing differentially expressed proteins (rows) in plasma from SAA group compared with HD group (Student’s *t*-test; fold change >2, Bonferroni adjusted *p*-value ≤0.05). **(E)** Dotplot showing enrichment pathways (ES > 0) of upregulated proteins in SAA patients compared with HD by gseGo analysis. ES, enrichment score; Humoral immune response*, humoral immune response mediated by circulating immunoglobulin; Adaptive immune response*, adaptive immune response based on somatic recombination of immune receptors built from immunoglobulin superfamily domains. **(F)** Cnetplot displaying protein network of seven pathways shown in panel E. SAA, severe aplastic anemia; LC-MS, liquid chromatography–mass spectrometry; PCA, principal component analysis; ES, enrichment score.

Plasma proteomics has gained much interest for identifying possible prognostic markers in disease conditions. We collected the plasma from 15 healthy donors and 14 SAA patients and performed untargeted proteomics and metabolomics LC-MS analyses (Figure 1A) as previously reported (Want et al., 2006, O’Maille et al., 2006; Yuan et al., 2012, Breitkopf et al., 2012). The clinical characteristics of patients are summarized in Table 1. We mixed four to five plasma samples as one sample for proteomics analysis.

**TABLE 1:** Clinical characteristics of the study population.

Characteristic	HD	SAA	*p*-Value
Age	—	—	—
Medium	30.33	29.73	0.8924
Range	17–54	20–64	—
Gender	—	—	0.8367
Female	7	6	—
Male	8	8	—
WBC, 10^9^/L	—	—	—
Average	—	1.71	—
Range	—	0.75–2.87	—
RBC, 10^12^/L	—	—	—
Average	—	2.03	—
Range	—	1.27–2.87	—
Hemoglobin, g/L	—	—	—
Average	—	62.31	—
Range	—	38–88	—
PLT, 10^12^/L	—		—
Average	—	16.36	—
Range	—	0–46	—
Patient type	—	—	—
Newly diagnosed	—	8	—
Remission	—	0	—
Non-remission	—	6	—
Drug	—		—
Anti-infective agents	—	7	—
Androgen	—	2	—
Ciclosporin	—	5	—
Eltrombopag	—	3	—
ATG	—	1	—
G-CSF	—	2	—

Note. HD, healthy donors; SAA, severe aplastic anemia; Remission, the patients who had been diagnosed and responded to the treatment; Non-remission, patients who had been diagnosed but did not respond to the treatment; ATG, antithymic globulin; G-CSF, granulocyte colony-stimulating factor; WBC, white blood cell; RBC, red blood cell; PLT, platelet count.

Plasma protein profile in SAA patients and HD is distinct and shows a good intra-group consistency by PCA and Pearson’s correlation analysis (Figure 1B; correlation coefficient >95%, Figure 1C). Of the 239 identified proteins, 49 had missingness and were removed from further analyses. Among the remaining proteins, 6 decreased and 15 increased proteins in SAA patients were observed (*p* < 0.05) (Figure 1D; Supplemental Material S1).

Upregulated proteins in SAA patients are enriched in immunoglobulin-mediated immune response (especially humoral immune response) and complement activation pathways by pathway enrichment analysis (Figure 1E). Moreover, complement molecules and serum amyloid A might participate in these pathways revealed by cnetplot analysis (Figure 1F).

### Metabolomics Analysis of Plasma and Bone Marrow Supernatant From Severe Aplastic Anemia Patients and Healthy Donors

**FIGURE 2:**
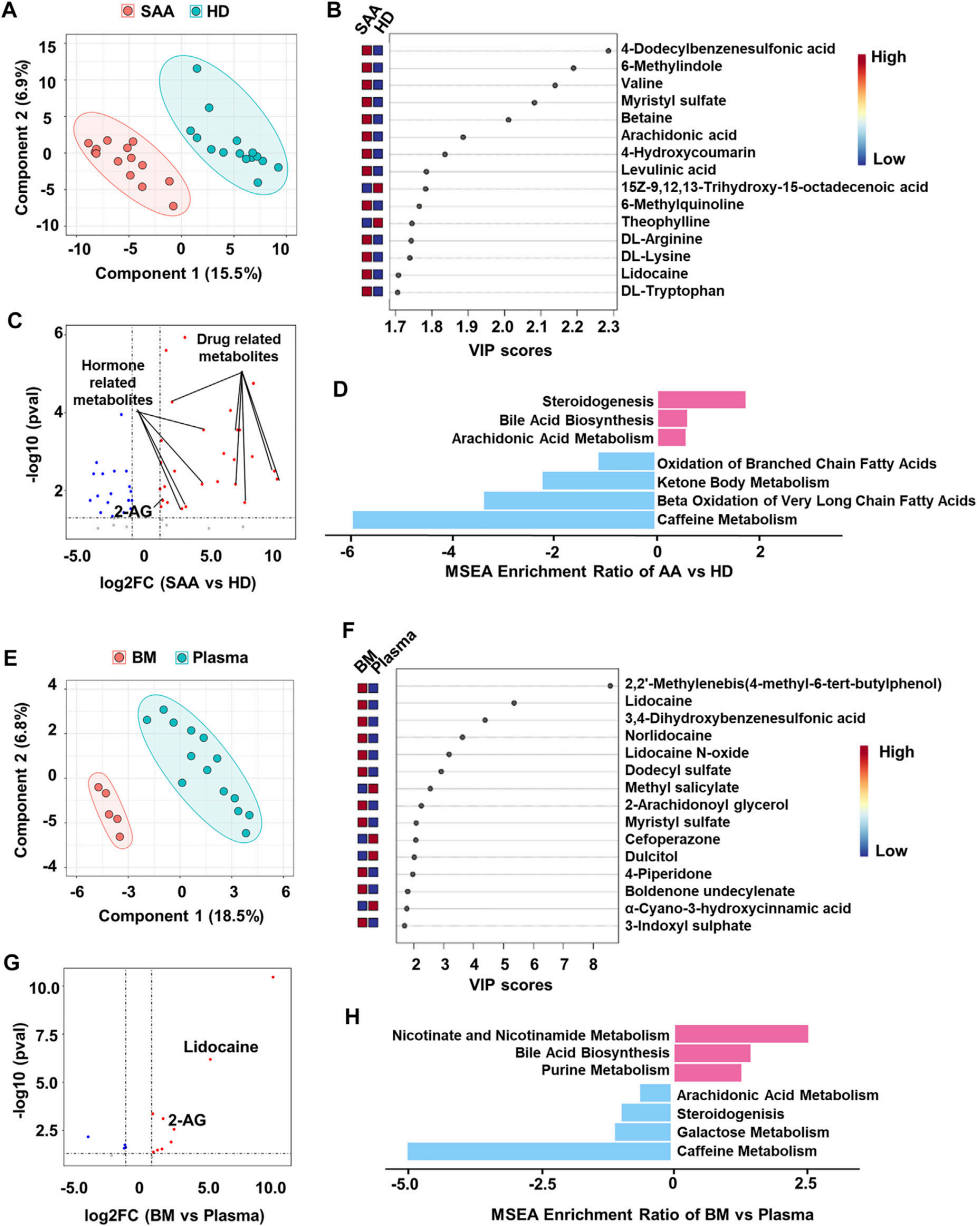
Metabolomics profiling of plasma from SAA patients and healthy donors. **(A)** PLS-DA plot showing the metabolic profiles of the SAA (pink dots, *n* = 14) and HD (green dots, *n* = 15) groups. **(B)** The variable importance in projection (VIP) score plot showing the top 15 PLS-DA metabolites that are different between SAA patients and HD. **(C)** Volcano plot showing differential metabolites (fold change >2, *p* < 0.05) between SAA patients and HD analyzed by MetaboAnalyst website. The upregulated metabolites are enriched with drug and hormone-related metabolites. **(D)** MSEA plot showing enriched pathways of upregulated (fold change >2) and downregulated (fold change <2) metabolites in the SAA group. **(E)** PLS-DA plot showing metabolic profiles of the BM supernatant (pink dots, *n* = 5) and plasma (green dots, *n* = 14) in SAA patients. **(F)** VIP score plot showing the top 15 PLS-DA metabolites that are different between BM supernatant and plasma. **(G)** Volcano plot showing differential metabolites (fold change >2, *p* < 0.05) between BM supernatant and plasma analyzed by MetaboAnalyst website. **(H)** MSEA plot showing enriched pathways of upregulated (fold change >2) and downregulated (fold change <2) metabolites in BM supernatant of SAA patients. SAA, severe aplastic anemia; PLS-DA, partial least squares discriminant analysis; HD, healthy donors; MSEA, metabolite set enrichment analysis; BM, bone marrow.

Several significantly changed proteins between SAA patients and healthy donors, such as SHBG, APOA1, and PGK1, were closely associated with the metabolic state of patients (Figure 1D). Therefore, we profiled metabolomics of plasma from SAA patients and HD. We identified 225 known metabolites for further analyses. SAA patients and HD have distinct metabolite profiles, which were analyzed by PLS-DA (Figure 2A). A total of 50 metabolites were significantly changed (22.2% of 225 metabolites detected; Figures 2B,C); 33 metabolites were obviously increased, and 17 metabolites were significantly decreased (Figure 2C, Supplemental Material S2). Among them, drug-related metabolites (cefoperazone, norlidocaine, lidocaine N-oxide, lidocaine, metronidazole, omeprazole sulfone, cilastatin, resmethrin, and boldenone undecylenate) were significantly increased in plasma of SAA patients as compared with those of healthy donors showed by variable importance in projection (VIP) scores and a volcano plot (Figures 2B,C, Supplemental Material S2). Interestingly, sex hormones such as progesterone and testosterone isocaproate were also elevated in the plasma of SAA patients (Figure 2C, Supplemental Material S2). Notably, upregulated metabolites in SAA patients were enriched in steroidogenesis, arachidonic acid metabolism, and bile acid biosynthesis, while downregulated metabolites were enriched in the oxidation of branched-chain fatty acids, ketone body metabolism, beta oxidation of very-long-chain fatty acids, and caffeine metabolism pathways by metabolite set enrichment analysis (MSEA) (Figure 2D).

BM is the main target in SAA. Therefore, we also collected the BM supernatant of SAA patients (*n* = 5) to profile the metabolome. PLS-DA plot perfectly clustered BM supernatant and plasma (Figure 2E), showing that the metabolites in BM supernatant significantly differed from those in plasma. Drug-related metabolites (lidocaine, lidocaine N-oxide, and norlidocaine) were also increased in BM supernatant (Figures 2F,G, Supplemental Material S3). Interestingly, we found that 2-arachidonoylglycerol (2-AG) was substantially increased in both plasma and BM supernatant (Figure 2C,E,F) of SAA patients, suggesting that the endocannabinoid system may participate in the disease development. Upregulated metabolites in BM supernatant are enriched in bile acid biosynthesis, nicotinate, and nicotinamide metabolism and purine metabolism pathways revealed by MSEA. Meanwhile, metabolites associated with steroidogenesis and arachidonic acid metabolism pathways were downregulated, as well as galactose metabolism and caffeine metabolism in BM supernatant (Figure 2H).

### Systemic Metabolic Change Is Associated With CD8+ T-Cell Activation in Aplastic Anemia

**FIGURE 3:**
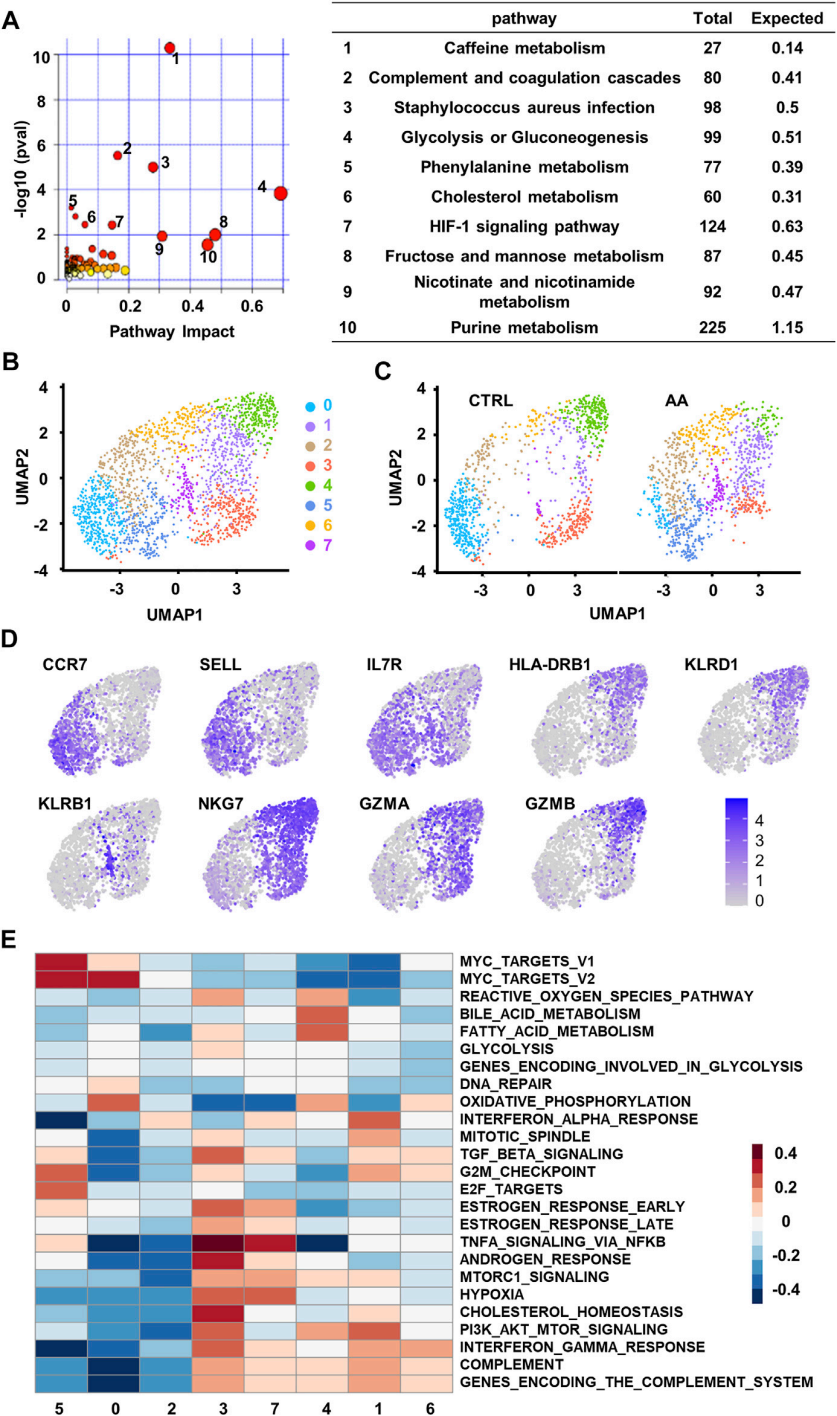
Single-cell transcriptional analysis reveals clusters of CD8^+^ T cells associated with plasma changes in AA patients. **(A)** Integrated join pathway analysis showing top 10 pathways of upregulated proteins and metabolites in SAA patients. **(B)** UMAP visualization of CD8^+^ T-cell clusters based on single-cell transcriptomes. Each dot represents a single cell; circles with different colors and numbered labels indicate cell clusters. **(C)** UMAP visualization of CD8^+^ T-cell clusters from healthy control (CTRL) and AA patients. **(D)** Feature plot showing marker gene expression in each cluster. **(E)** GSVA heatmap showing hallmark pathways enriched in different clusters. AA, aplastic anemia; SAA, severe aplastic anemia; UMAP, Uniform Manifold Approximation and Projection; GSVA, gene set variation analysis.

To fully understand the functional role of these changes in the plasma of SAA patients, we combined the plasma proteomics and metabolomics data by joint pathway analysis (Basu et al., 2017, Duren et al., 2017) by analyzing upregulated proteins and metabolites. The results of the enriched 119 pathways were simultaneously plotted to screen pathways in terms of hypergeometric test *p*-values and pathway impact (Figure 3A). We found that the top ten pathways by *p*-value (top six) or impact (top four) were indicated the following pathways: glycolysis or gluconeogenesis, fructose, and mannose metabolism, purine metabolism, caffeine metabolism, nicotinate and nicotinamide metabolism, staphylococcus aureus infection, complement and coagulation cascades, HIF-1 signaling, cholesterol metabolism, and phenylalanine metabolism pathways (Figure 3A). Alteration in these pathways in SAA plasma provides clues that the coordination changes in proteins and metabolites may impact the system energy metabolism and immune response of SAA patients.

To find a possible link between systemic metabolic changes and CD8^+^ T-cell activation in AA patients, we further analyzed single-cell transcriptome data (GSE145669) of CD8^+^ T cells from AA patients and healthy controls (Zhu et al., 2021, Lian et al., 2021). Dimension reduction and unsupervised clustering classified CD8^+^ T cells into eight groups with distinct gene expression patterns (Figure 3B). Cells in clusters 0, 2, and 5 highly expressed the markers associated with the naïve T-cell phenotype, such as *CCR7*, *SELL*, and *IL7R*; cells in clusters 1, 3, 4, and 6 highly expressed the markers associated with memory or effector phenotypes, such as *HLA-DRB1*, *KLRD1*, *NKG7*, *GZMA*, and *GZMB*; and cells in cluster 7 highly expressed the genes associated with long-lived memory precursor cells (Kaech et al., 2003, Tan et al., 2003), such as *KLRB1* and *IL7R* (Figure 3C).

Among the eight clusters, cells in clusters 1, 5, and 7 increased in AA patients (Figure 3D), which enriched genes involved in cell proliferation and survival, such as cell cycle phase, MYC target, and PI3K/AKT/MTOR pathways. They also express genes associated with proinflammation pathways that are involved in AA, such as TNFα signaling (Figure 3E). These results suggest that CD8^+^ T cells in AA patients were different from healthy controls in both phenotype and intrinsic pathways, which may be responsible for the systemic metabolic changes in AA.

### CD38^+^CD8^+^ T Cells Are Highly Active and Increase in Aplastic Anemia Patients

**FIGURE 4:**
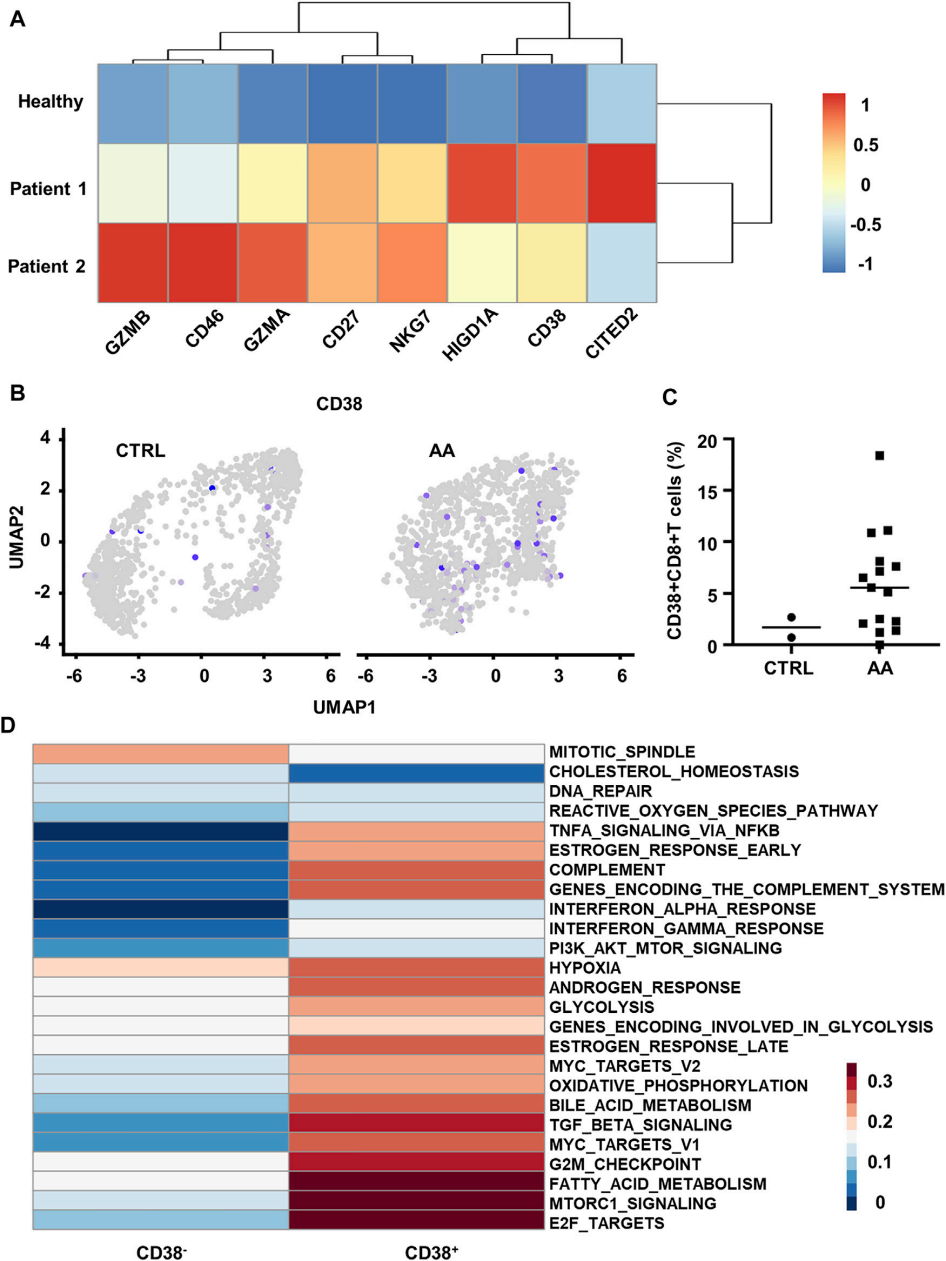
Transcriptional and scRNA-seq analysis reveals increased CD38^+^CD8^+^ T cells in AA patients. **(A)** Heatmap showing differential genes between BM T cells from healthy control (GSM87365), Patient 1 (SAA patient, GSM87341), and Patient 2 (SAA patient, GSM87360) in microarray data (GSE3807). **(B)** Feature plot showing the expression of CD38 in CTRL and AA. **(C)** Scatter plot showing the proportion of CD38^+^CD8^+^ T cells (expression value >0) in CD8^+^ T cells from CTRL (*n* = 2) and AA patients (*n* = 15). **(D)** GSVA heatmap showing hallmark pathways enriched in CD38^+^ and CD38^−^ cells. CD38^+^ cells were defined by the expression level of CD38 gene. AA, aplastic anemia; BM, bone marrow; CTRL, healthy control; GSVA, gene set variation analysis.

We further discovered the elevated expression of T-cell activation (CD38, GZMB, GZMA, CD27, and NKG7) genes in BM T cells from AA patients by analyzing microarray data (GSE3807) (Franzke et al., 2006, Geffers et al., 2006) (Figure 4A). Previous reports showed that CD38 drives T-cell proliferation, cytokine secretion, and activation (Muñoz et al., 2008, Mittelbrunn et al., 2008). Also, CD38 is closely related to the metabolic state and regulates T cells' fate due to its multifunctional enzymatic activity such as NAD/NMNase (Hogan et al., 2019, Chini et al., 2019).

On this basis, we hypothesized that CD38 might be involved in the pathogenic process of CD8^+^ T cells in SAA. We defined CD38^+^ cells by the expression value of CD38 > 0 in the single-cell transcriptome data (GSE145669) and observed that CD38^+^CD8^+^ T cells are mainly grouped within clusters 1, 5, and 7 (Figure 4B). Moreover, we found that CD38^+^CD8^+^ T cells were significantly increased in patients (Figure 4C).

Further, we compared the pathway variation between CD38^+^CD8^+^ T cells and CD38^−^CD8^+^ T cells within clusters 1, 5, and 7. CD38^+^CD8^+^ T cells express genes enriched with the pathway participated in SAA by GSVA, such as TNF-α signaling, indicating that CD38^+^CD8^+^ T cells might be important in SAA (Figure 4D). CD38^+^CD8^+^ T cells also express hallmark genes involved in hypoxia, glycolysis, fatty acid metabolism, and complement pathways (Figure 4D). Moreover, we observed that T-cell microarray data displayed increased hypoxia-associated gene expression (*CD46*, *CITED2*, and *HIGD1A*) in AA patients (Figure 4A), which further demonstrated that CD38^+^CD8^+^ T cells are activated and undergo metabolic changes in AA. These data indicated that CD38^+^CD8^+^ T cells might be important in disease development.

### Bone Marrow Infiltration of CD38^+^CD8^+^ T Cells in Murine Aplastic Anemia Model

**FIGURE 5:**
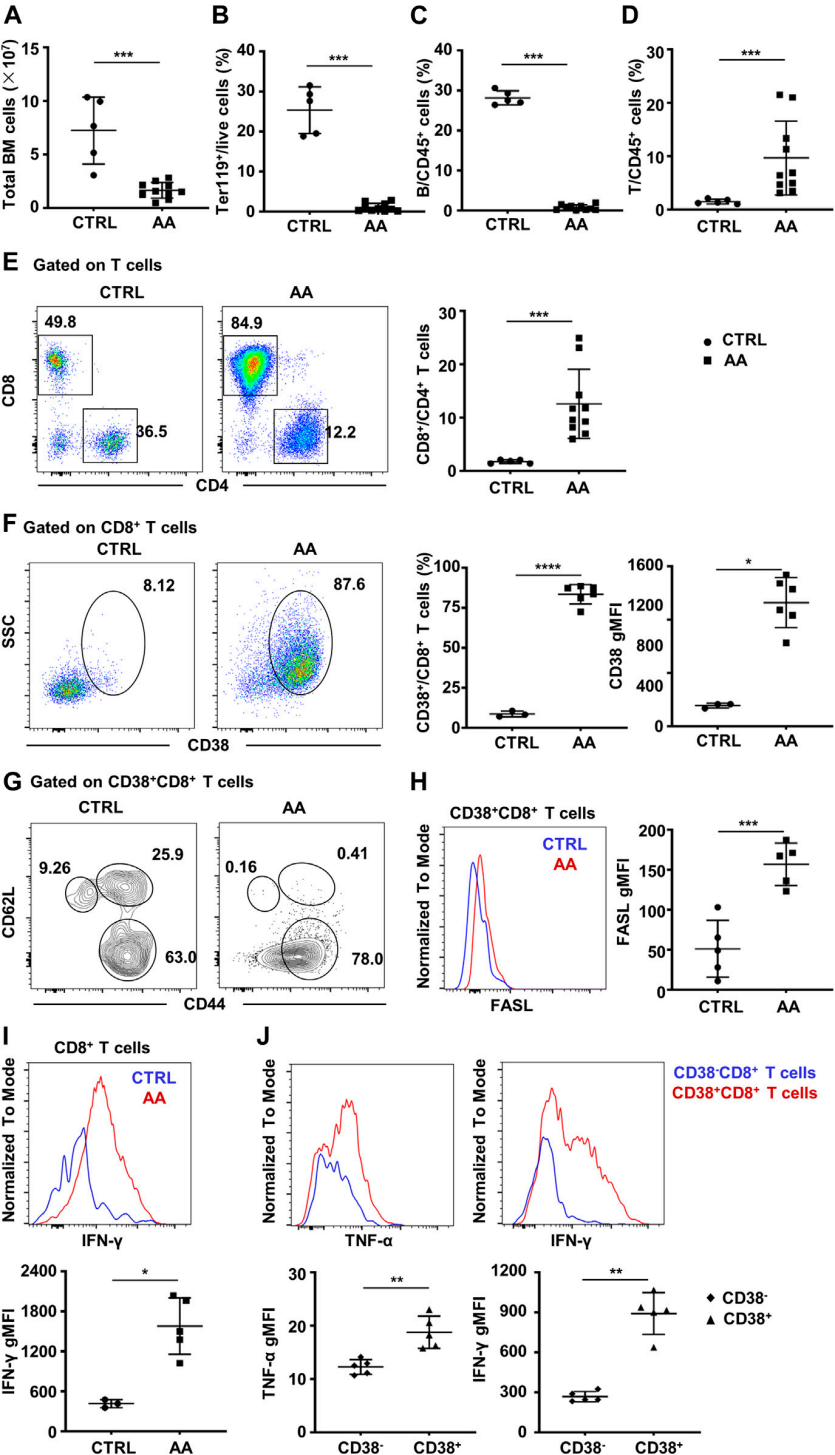
CD38^+^CD8^+^ T cells increase in BM of murine AA model. **(A)** BM total nucleated cell numbers of AA model (*n* = 10) and CTRL mice (*n* = 5). Percentages of Ter119^+^ cells **(B)**, B cells **(C)**, and T cells **(D)** of AA model (*n* = 10) and CTRL mice (*n* = 5); Mann–Whitney test. **(E)** Representative flow cytometry result and of the percentages of CD8^+^ and CD4^+^ T cells and statistical analysis of CD8^+^/CD4^+^ T cell ratio between AA model (*n* = 10) and CTRL mice (*n* = 5); Mann–Whitney test. **(F)** Representative flow cytometry result of CD38 expression on BM CD8^+^ T cells and statistical analysis of CD38^+^CD8^+^ T cells (Student’s *t*-test) and geometry MFI of CD38 (right, Mann–Whitney test) on CD8^+^ T cells in AA (*n* = 5) and CTRL (*n* = 3) mice. **(G)** The expression of CD44 and CD62L on CD38^+^CD8^+^ T cells. **(H)** Representative flow cytometry result **(left)** and statistical analysis **(right)** of FASL secretion ability by CD38^+^CD8^+^ T cells in AA model (*n* = 5) and CTRL mice (*n* = 5). Student’s *t*-test. **(I)** Representative flow cytometry result **(up)** and statistical analysis **(down)** of IFN-γ secretion ability by CD8^+^ T cells in AA (*n* = 5) and CTRL (n = 3) mice; Mann–Whitney test. **(J)** Representative flow cytometry result and statistical analysis of IFN-γ and TNF-α secretion ability by CD38^+^CD8^+^ T cells and CD38^−^CD8^+^ T cells in AA model (*n* = 5); Mann–Whitney test. *p < 0.05, **p < 0.01, ***p < 0.001. BM, bone marrow; AA, aplastic anemia; CTRL, healthy control; MFI, mean fluorescence intensity.

To demonstrate the pathogenic role of CD38^+^CD8^+^ T cells in AA, we then constructed a murine immune-mediated AA model by infusing C57BL/6 lymphocytes into CByB6F1 mice, which induces AA symptoms such as hypocellular BM and lymphocyte infiltration in the BM (Krenger et al., 2000, Rossi et al., 2000). We observed a significant decline in the total number of BM cells (Figure 5A), as well as a decreased percentage of Ter119^+^ cells (Figure 5B) and B cells (Figure 5C). However, the percentage of T cells (Figure 5D) was significantly increased, and CD8^+^ T cells were predominant in the BM (Figure 5E).

Consistent with single-cell transcriptome data, CD38^+^CD8^+^ T cells significantly increased in AA mice, as well as the expression and MFI of CD38 (Figure 5F). Moreover, most of CD38^+^CD8^+^ T cells were CD62L^−^CD44^+^ (Figure 5G), especially in AA mice. It has been evidenced that CD8^+^ T cells usually achieve hematopoietic cell injury through the Fas/FasL pathway (Young et al., 2006, Calado et al., 2006). Hence, we analyzed the level of FasL on CD38^+^CD8^+^ T cells between AA and CTRL. As expected, the expression of FasL was significantly higher in AA mice, which was consistent with a previous report (Figure 5H). Also, BM CD8^+^ T cells from AA mice have significantly elevated IFN-γ secretion ability (Figure 5I). We also detected the secretion of IFN-γ and TNF-α by CD38^+^CD8^+^ T cells and CD38^−^CD8^+^ T cells in the BM of the induced AA murine model. We found that CD38^+^CD8^+^-derived T cells secreted more IFN-γ and TNF-α, indicating that CD38^+^CD8^+^ T cells might be the more potent pathogenic immune cells during AA development (Figure 5J). These data suggest an activated phenotype of CD38^+^CD8^+^ T cells in the BM of AA.

## Discussion

In this work, we show that the plasma proteome and metabolome of SAA patients differ from those of healthy donors in pathways involved in the T-cell activation, such as glycolysis or gluconeogenesis, cholesterol metabolism, complement, and HIF-1 signaling pathways. Moreover, we find that CD8^+^ T cells from AA patients, especially the CD38^+^CD8^+^ T cell subset that is increased in AA patients and murine AA model, are enriched with these pathways. CD38^+^CD8^+^ T have higher proinflammatory and proliferative capacity, indicating they may contribute to the pathologic progression in AA.

The current studies on CD38^+^CD8^+^ T cells mainly focus on their role in infectious diseases (Chun et al., 2004, Justement et al., 2004; Resino et al., 2004, Bellón et al., 2004). In autoimmune diseases, the role of CD38 is still obscure. CD38 can impair the cytotoxic function of CD8^+^ T cells in systemic lupus erythematosus (SLE), leading to an increased susceptibility of SLE patients to infections (Katsuyama et al., 2020, Suarez-Fueyo et al., 2020). A highly proliferative and unconventional CD38^+^CD45RA^+^T-BET^−^ IL-10-producing polyclonal T-cell population is identified in autoimmune lymphoproliferative syndrome, which is tightly controlled by FAS and CTLA4 and maintained by mammalian target of rapamycin (mTOR) and STAT3 signals (Maccari et al., 2021, Fuchs et al., 2021). Our analysis shows that CD38^+^CD8^+^ T cells infiltrate into the BM of AA patients and murine AA model. The infiltrated CD38^+^CD8^+^ T cells may destroy BM, indicating the pathogenic role of CD38 in AA. Indeed, CD38^+^CD8^+^ T cells exhibit higher potential in survival and proliferation and promote inflammation. Complement also shows enrichment in CD38^+^CD8^+^ T cells, suggesting the involvement of complement system, as for intracellular complement tightly related to effector differentiation (Liszewski et al., 2013, Kolev et al., 2013). However, the mechanisms need further investigation.

Interestingly, we found that hypoxia is one of upregulated hallmark gene sets in CD38^+^CD8^+^ T cells. Cell metabolic status and surrounding niche are critical factors for CD8^+^ T-cell activation and function (Xie et al., 2020, Li et al., 2020). Oxidative phosphorylation (OXPHOS), glycolysis, glutaminolysis, and fatty acid metabolism are closely related to the survival, proliferation, and memory formation of CD8^+^ T cells (Zhang and Romero, 2018). Besides, hypoxia plays an important role in regulating CD8^+^ T cells' function and fate decisions (Zhang and Romero, 2018). BM is hypoxic under normal conditions (Parmar et al., 2007, Mauch et al., 2007). Studies show that exacerbating hypoxia occurs in diseased BM (Mortensen et al., 1998, Jensen et al., 1998). Interestingly, CD38^+^CD8^+^ T cells express genes involved in hallmarks of hypoxia. Considering its possible relationship with hypoxia (Hoff et al., 2013, Maschmeyer et al., 2013; Zhang et al., 2020, Li et al., 2020), CD38 may play an important role in the activation of CD8^+^ T cells in the BM of AA patients.

Androgen has been used for the treatment of AA (Bär et al., 2015, Huber et al., 2015), while the changes in hormones in SAA patients are unknown. Our data show that steroidogenesis is upregulated in SAA. Also, hormone-related metabolites are significantly increased in plasma and BM supernatant from SAA patients, such as progesterone and testosterone isocaproate, which may reflect the imbalance of hormones in AA. Moreover, CD38^+^CD8^+^ T cells exhibit higher ability in response to estrogen or androgen. These data coincide with the complex network of cholesterol metabolism and steroidogenesis. Besides, 2-AG is increased in both plasma and BM supernatant from SAA patients. Recent research reports the important role of 2-AG receptor CB2R in regulating activation of CD8^+^ T cells during graft-versus-host disease (GVHD) development (Yuan et al., 2021, Zhou et al., 2021), indicating that the endocannabinoid system may participate in AA pathogenesis.

However, there are limitations to this study. First, more samples and another patient cohort are needed to validate findings. Second, mechanisms of how these molecules and pathways are involved in AA pathogenesis, which need further investigation. Nevertheless, our study provides a proteome and metabolome map of AA plasma and BM and reveals several pathways that are associated with CD8^+^ T-cell activation in SAA. These findings will promote our understanding of AA pathogenesis, therefore providing potential therapeutic targets for SAA treatment.

## Data Availability

The original contributions presented in the study are publicly available. This data can be found here: IPX0003580000.
